# A viral mimic increases body temperature but does not affect mass or inflammation in a wild frugivorous bat

**DOI:** 10.1098/rsos.262283

**Published:** 2026-11-11

**Authors:** Alexis M. Heckley, Valeriia Bohodist, Camilo A. Calderón-Acevedo, Alona Prylutska, Maryna Yerofeieva, Kateryna Zemliana, Rachel A. Page, Ralph Simon, Daniel J. Becker

**Affiliations:** ^1^ School of Biological Sciences, University of Oklahoma, Norman, OK, USA; ^2^ Center for Animal Behavior, Smithsonian Tropical Research Institute, Gamboa, Panama; ^3^ Ukrainian Bat Rehabilitation Center, Ukrainian Independent Ecology Institute, Kharkiv, Ukraine; ^4^ Finnish Museum of Natural History, University of Helsinki, Helsinki, Finland; ^5^ H.S. Skovoroda Kharkiv National Pedagogical University, Kharkiv, Ukraine; ^6^ Max Planck-Smithsonian Center for Sensory Ecology and Social Evolution, Panama; ^7^ Falz-Fein Biosphere Reserve Askania Nova, Ukraine; ^8^ Behavioral Ecology and Conservation Lab, Nuremberg Zoo, Nuremberg, Germany; ^9^ Department Artificial Intelligence in Biomedical Engineering, Friedrich-Alexander-Universität Erlangen, Erlangen, Germany

**Keywords:** Chiroptera, antiviral, poly(I:C), ecoimmunology, innate immunity, sickness

## Abstract

The acute phase response is a component of innate immunity that helps fight infections. Understanding variation in this response could be critical in bats, which can be asymptomatic hosts of pathogens that cause disease in other animals. Although bats are most famously tolerant of some viruses, research on the bat acute phase response has focused predominantly on bacterial antigens. To improve understanding of bat viral responses, we challenged wild Seba's short-tailed bats (*Carollia perspicillata*) with a viral mimic (polyinosinic:polycytidylic acid (polyI:C)). We injected nine bats subcutaneously with 2 mg kg^−1^ of polyI:C (*n* = 3), 5 mg kg^−1^ of polyI:C (*n* = 3) or phosphate buffered saline (*n* = 3). Over the next 24 hours, we measured body temperature hourly and collected body mass and blood smears for leucocyte counts every 4 hours. Regardless of dose, polyI:C-challenged bats had higher body temperatures compared to control bats but did not exhibit leucocytosis or reduced body mass. These preliminary findings improve understanding of how wild bats physiologically respond to viral challenges. Moreover, in showing that as little as 2 mg kg^−1^ of polyI:C can induce a febrile response, our study provides a framework to facilitate future investigations into causes and consequences of wild bat viral responses.

## 1. Introduction

The acute phase response is one of the first lines of defence against pathogens [1]. Mounting this immune response is energetically costly and manifests as a suite of behavioural and physiological symptoms that include but are not limited to lethargy, fever and body mass loss [2,3]. Because these ‘sickness’ effects can trade-off with other ecologically important processes, such as foraging, mating or territorial defence, these responses can be deleterious to hosts [4]. Yet despite its potential costs, sickness has also been hypothesized to be adaptive [5]. The behavioural and physiological changes that characterize sickness permit energy allocation towards fighting infections and thus can be ultimately advantageous to hosts. Moreover, sickness can also shape population- and community-level processes. For instance, symptoms such as lethargy can affect contact rates between individuals, altering parasite transmission within and between species (e.g. [5]). As such, the acute phase response is critically important for determining wildlife infection outcomes.

Controlling or predicting pathogen transmission, or mitigating deleterious effects of pathogens on hosts, therefore requires a robust understanding of sickness. Yet sickness is highly variable, and many factors can underlie this variation. For instance, life-history strategies can sometimes determine the extent to which sickness manifests [2], and different pathogen taxa can produce distinct sickness responses [6]. Comparative studies could help untangle the ecological and evolutionary factors that underlie this variation. However, sickness research has primarily been rooted in laboratory systems, and work within wild systems has emphasized only certain antigen types. An essential first step in improving understanding of sickness is therefore to characterize variation in the acute phase response in wild animals. This endeavour is particularly critical in potentially zoonotic systems, where variation in sickness could inform spillover prevention strategies, and in host groups where sickness is not always presented as classically expected.

Bats (order: Chiroptera) are a particularly interesting host group for investigating variation in the acute phase response, because they can harbour pathogens that cause clinical disease in other animals, including humans, yet often while remaining asymptomatic [7–9]. For instance, whereas Marburg virus (MARV) causes severe haemorrhagic fever in humans, Egyptian fruit bats (*Rousettus aegyptiacus)* inoculated with MARV do not show signs of clinical disease [10,11]. A notable absence of disease also occurs for other bat species inoculated with other viruses, including Jamaican fruit bats (*Artibeus jamaicensis*) with Middle East respiratory syndrome coronavirus [12] and *Pteropus* species with both Hendra virus (HeV) and Nipah virus (NiV) [13]. Similarly, when experimentally challenged with antigens that mimic bacterial infections, bats do not always show characteristic features of the acute phase response, such as fever and leucocytosis (e.g. [14,15]); however, these and other sickness responses do sometimes occur (e.g. reduced food intake, altered foraging and social behaviour, increased resting metabolic rates and body mass losses; [14–25]). Understanding the causes and consequences of variation in the acute phase response across bats is foundational for research on the ecology and evolution of infections and could facilitate development of novel therapies to treat infections in humans and other wildlife [8,26].

Experimental immune challenges are desirable for studying the acute phase response in wildlife, because they isolate the effects of the immune response without any possible confounding effects of an actual pathogen [27]. Moreover, in contrast to observational work, these manipulations provide causal insight into how immune responses affect downstream processes (e.g. physiological responses). In bats specifically, where experimental infections can be prohibitively challenging for logistical and regulatory reasons, immune challenge in the wild can serve as a more tractable system for studying infection *in vivo* [28]. However, most experimental research in bats and other animals has focused on immunological responses to simulated bacterial infections using lipopolysaccharide (LPS), a Gram-negative bacterial cell wall component [2]. This work has advanced understanding of the mechanisms underlying variable host responses to bacterial infections, revealing substantial inter- and intra-specific differences in the acute phase response driven by organismal features (e.g. life-history variation) [29] and environmental conditions (e.g. ambient temperature) [25]. LPS challenges have also shed light on consequences of the acute phase response, such as how sickness behaviour can alter pathogen transmission dynamics (e.g. [30]). Given the eco-evolutionary, biomedical and conservation importance of many bat-harboured viruses (e.g. MARV, HeV, NiV, lyssaviruses), a need exists for more focused work on how viral challenges (rather than bacterial challenges) affect the acute phase response in bats.

Polyinosinic:polycytidylic acid (polyI:C) is a double-stranded RNA virus analogue that initiates immune responses similar to those triggered by viral infections [31]. In bats, *in vitro* polyI:C challenges have revealed diverse effects on gene expression and protein abundance (e.g. [32–36]). To provide some examples, bat cell lines transfected with polyI:C upregulate interferon signalling and interferon-stimulated gene transcription, both of which work to control viral infections (e.g. [32,37]). Moreover, polyI:C-transfected black flying fox (*Pteropus alecto*) cell lines upregulate glycolysis-associated proteins, suggesting increased energy metabolism, and downregulate ribosomal-associated proteins, possibly to limit viral replication [37]. As a final example, macrophages of greater mouse-eared bats (*Myotis myotis*) challenged with polyI:C show both proinflammatory and anti-inflammatory responses, which are hypothesized to simultaneously control infections while minimizing damage to host cells [38].

To date, only a small number of studies have experimentally challenged wild bats *in vivo* with polyI:C [39–42]. For example, with *M. myotis*, a Eurasian vespertilionid species, polyI:C treatment did not induce plasma oxidative damage [39]. Additionally, polyI:C treatment in this same bat species did not affect fever, leucocytosis or body mass, although challenged bats increased their neutrophil-to-lymphocyte ratio [41]. Some of these findings contrast with work on the great fruit-eating bat (*Artibeus lituratus*), a phyllostomid distributed across the Greater Antilles, Central America and northern South America, where polyI:C caused bats to lose body mass and develop fever in addition to increasing resting metabolic rate [42]. Much like responses to LPS, these polyI:C studies suggest a high extent of diversity in virus-associated acute phase responses. Yet given the scarcity of this work, additional studies in other species are critically needed to improve understanding of viral-associated acute phase responses in wild bats [26].

Here, we investigated how simulated viral challenges with polyI:C affect the acute phase response in Seba's short-tailed bat (*Carollia perspicillata*), a small-to-medium sized phyllostomid bat species that is likewise distributed across Central and South America [43]. *Carollia perspicillata* is one of the most heavily studied Neotropical bat species and is emerging as an important model for studies of bat–virus interactions (e.g. [44,45]). Following bacterial challenge with LPS, *C. perspicillata* lose body mass and sometimes exhibit leucocytosis and increased body temperature [14,22,25]. A recent study demonstrated that *C. perspicillata* cell lines respond to polyI:C by upregulating particular interferons and interferon-induced proteins [45]. However, no studies have investigated how *C. perspicillata* respond to viral challenges *in vivo*. It therefore remains unknown how antiviral physiological responses manifest in this species at the organismal level and in the wild. To address this knowledge gap, we injected wild-caught *C. perspicillata* in Panama with two different polyI:C doses or a phosphate-buffered saline (PBS) control and recorded changes to body temperature, body mass and total leucocyte counts over a 24-hour period. This approach allowed us to address three objectives: (i) establish whether polyI:C induces an antiviral response *in vivo* in this species; (ii) characterize how this viral mimic affects *C. perspicillata* sickness physiology; and (iii) provide recommendations on appropriate doses for studying *C. perspicillata* antiviral responses to facilitate future work. Our preliminary results show that, regardless of dose, *C. perspicillata* challenged with polyI:C develop fever but do not exhibit body mass losses or changes to leucocyte counts. These results improve understanding of how these bats respond to viral antigens while also providing a framework for future researchers conducting experimental immune challenges in wild bats.

## 2. Material and methods

### 2.1. Bat capture and immune challenge

On 12 February 2026, we captured nine adult male *C. perspicillata* using mist nets outside a large roost in Gamboa, Parque Nacional Soberanía, Panama. We focused only on males because most captured females during this time were pregnant. Upon capture, we transferred bats to a large outdoor flight cage at the Smithsonian Tropical Research Institute, where they were housed together within a smaller tent (60 × 40 × 40 cm). We injected each bat with a thermo-sensitive passive integrated transponder (PIT) tag (Biotherm 13 PIT tag, Biomark) for individual identification and body temperature monitoring. Over the next several nights of acclimation, bats were provided water and banana ad libitum. All bats were captured at the same time, at the same place, and held in the same general conditions leading up to and during the trial (see below).

The experiment began slightly less than three days after capture (approx. 68 hours). Starting at 19.00, we removed bats from their tent and recorded mass with a Pesola scale (to the nearest 0.1 gram) and body temperature by scanning the thermo-sensitive PIT tags. Using 26- or 27-gauge sterile needles, we drew a small amount of blood (less than 10 µl) by lancing the propatagial vein. We collected blood with heparinized capillary tubes and transferred a drop to a clean glass slide to make a thin blood smear. We next injected each bat subcutaneously with one of either a low-dose of polyI:C (2 mg kg^−1^; *n* = 3 bats), a high-dose of polyI:C (5 mg kg^−1^; *n* = 3 bats), or PBS (using the equivalent volume as a 5 mg kg^−1^ polyI:C dose; *n* = 3 bats). While injecting, we alternated through treatments rather than injecting all three bats within one treatment group first. While we acknowledge the limitations posed by this small sample size, we selected this number of individuals for the logistical and welfare concerns associated with keeping bats in captivity and conducting repeated blood draws over the study period.

Following injections, we immediately placed bats into small tents (*n* = 9 tents; each bat held individually), still within the outdoor flight cage. Within each tent, we provided 60 g of banana. Six of the tents were 60 × 40 × 40 cm, and the other three tents were 30 × 30 × 30 cm. Every hour following the injections (for 24 hours), we recorded body temperature by scanning each bat with a portable PIT tag reader (GPR Plus, Biomark). We aimed to be as minimally disruptive as possible, by holding the PIT tag reader to the side of the tent to minimize disturbance. Every 4 hours following the challenges, we removed bats from their tents, re-measured mass and collected blood to prepare another thin blood smear. The total volume of blood collected over this 24-hour period represented substantially less than 1% body mass [46]. After the trial was complete, bats were released at their capture location.

The 24-hour duration was selected based on previous studies with non-pathogenic antigens that report leucocytosis and body mass losses in bats less than or equal to 24 hours following LPS injection [22,24]. While studies on polyI:C in bats are less common, *A. lituratus,* a species within the same family as *C. perspicillata* (Phyllostomidae), lost mass within only 6 hours following polyI:C treatment [42]. Also within 6 hours, early work with mice found an increase in circulating white blood cells following intraperitoneal polyI:C injection [47]. This past work establishes 24 hours as a relevant timeline for detecting initial immune responses to these challenges.

### 2.2. Haematological analysis

Slides were air dried in the field and then stained with Wright–Giemsa (Quick III Set, Astral Diagnostics Inc.). We used blood smears to estimate total white blood cell counts [48]. Specifically, we used a light microscope to estimate the mean number of leucocytes from 10 fields of view at 400× magnification. We manually performed these counts rather than relying on a Neubauer chamber given time-related constraints while in the field, and our methods are consistent with other bat haematology work (e.g. [49]). Only monolayers were analysed, and one observer (A.M.H.) performed all white blood cell counts.

### 2.3. Statistical analysis

We conducted all analyses in R v 4.3.1 [50]. To test how immune challenges affect *C. perspicillata* body temperature (°C), body mass (g) and total white blood cell counts, we fitted three generalized additive mixed models (GAMMs)—one for each physiological measurement—using the *mgcv* package [51]. Rather than using the raw physiological measurements, we instead calculated the change from baseline (i.e. the measurement taken immediately prior to injections, at hour zero) for each sampling event (see the supplementary material, figures S1–S3, for raw physiological data). Stated another way, for each individual and physiological metric, we subtracted the individuals’ initial measurement from all measurements; the final values therefore represent the change in that physiological metric relative to the individual's pre-injection baseline. This metric is the most appropriate for monitoring within-individual physiological changes, as our sample size precluded the use of random slopes in our GAMMs (i.e. random factor smooths) [52,53]. In each model, we included treatment (PBS, low-dose polyI:C, high-dose polyI:C) as an ordered factor as a fixed effect, along with smoothed effects of time and treatment–time interactions using thin plate regression splines [54]. We also included bat identity as a random intercept to account for repeated measurements from the same individuals. All GAMMs were fitted using restricted maximum likelihood and used Gaussian errors. Model diagnostic outputs generated using *mgcv* are provided in the supplementary material, table S1 and figures S3–S6. To determine the extent to which total variation in the data could be attributed to among-individual differences, we calculated repeatability for each response variable (i.e. temperature, mass, and white blood cell changes from baseline) using the *ICC* package [55]. Finally, we conducted a post-hoc power analysis using the *pwr* package, assuming a moderate effect size (*F* = 0.25), 80% power and a traditional *α* cut-off (0.05) [56].

## 3. Results

We found that polyI:C treatment significantly increased *C. perspicillata* body temperature and that temperature increased linearly with dose (i.e. PBS < low dose < high dose; figure 1; table 1). Within the low-dose group, temperature changes differed non-linearly over time (estimated degrees of freedom (EDF) = 2.50), whereas we did not detect a time effect for the high-dose group (figure 1; table 1). This GAMM explained 57% of the deviance and 55% of the variance in temperature (i.e. *R*^2^ = 0.55).

**Figure 1.**
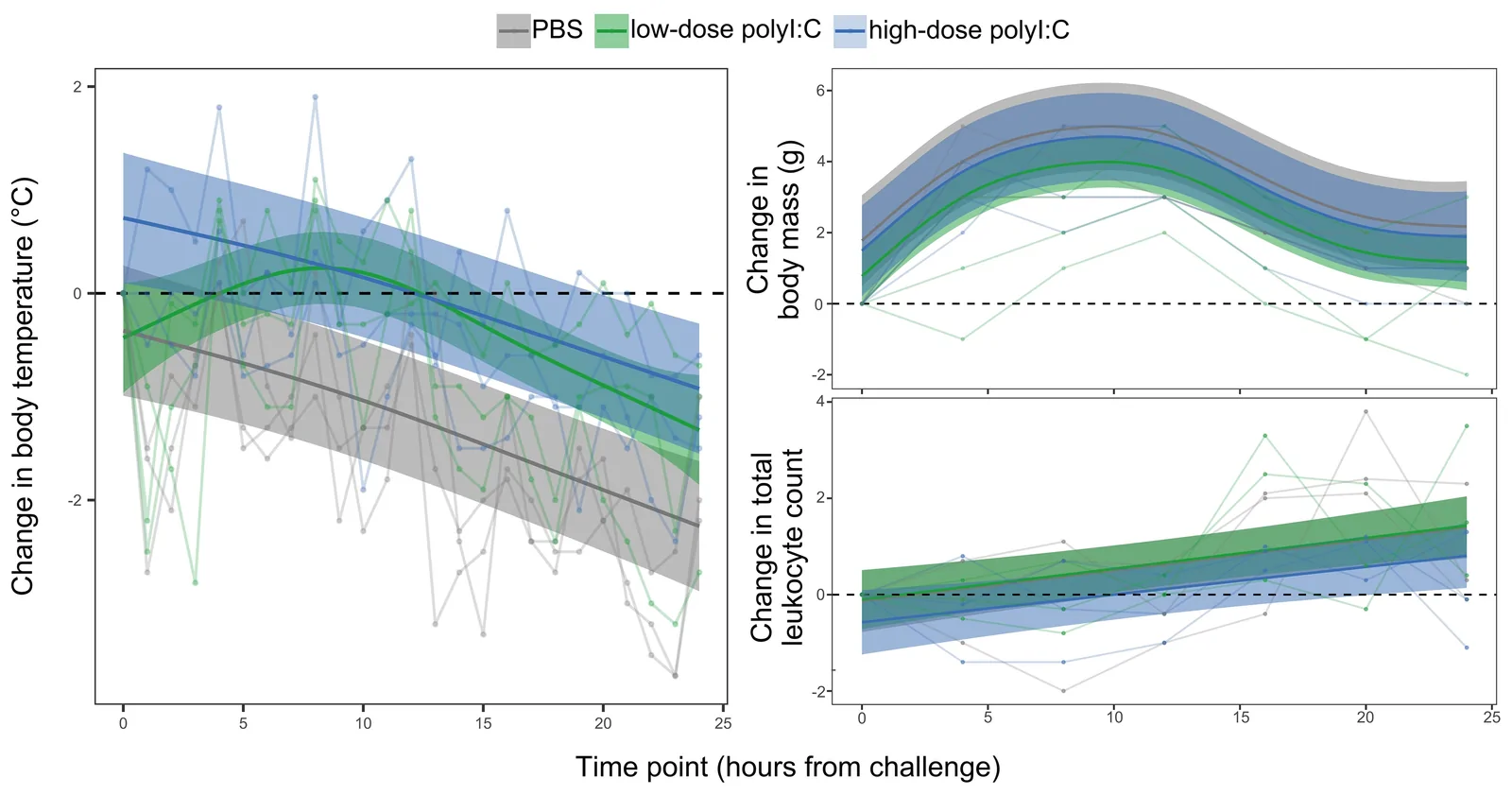
Effect of time on change in body temperature, body mass and total leukocyte count from baseline (i.e. measurements taken before injection, at time point 0). The trendlines with 95% confidence intervals were estimated from the GAMM, and the data points reflect raw data (adjusted to change-from-baseline). The data, trend lines and ribbons are coloured by treatment, and the horizontal dashed line reflects baseline. Because the response variables represent change-from-baseline, note that in all cases where *x* = 0, *y* = 0. As such, trend lines above or below *y* = 0 do not reflect higher or lower baseline measurements. Further, because the change in physiology represents a change relative to self at baseline, data points above the horizontal dashed line reflect a temperature, mass or leucocyte count increase from baseline. Accordingly, data points below the horizontal dashed line reflect a temperature, mass or leucocyte count decrease from baseline.

**Table 1.** Effects of treatment (as an ordered factor) on change from pre-injection baseline for each physiological measurement. (Effects are presented as model coefficients or estimated degrees of freedom (EDF) and test statistics. All GAMMs include a random intercept of bat identity.)

response	term	*β*	*t*	EDF	*F*	*p*
body temperature	intercept	−0.90	−7.73			<0.001
	treatment (linear)	0.86	4.27			<0.001
	treatment (quadratic)	−0.25	−1.25			0.213
	s(time point)			1.39	11.39	<0.001
	s(time point): low-dose polyI:C			2.50	2.85	<0.001
	s(time point): high-dose polyI:C			0.40	0.15	0.12
body mass	intercept	1.76	7.45			<0.001
	treatment (linear)	−0.20	−0.49			0.62
	treatment (quadratic)	0.70	1.71			0.09
	s(time point)			4.03	19.05	<0.001
	s(time point): low-dose polyI:C			0.00	0.00	0.24
	s(time point): high-dose polyI:C			0.00	0.00	0.77
leucocyte count	intercept	0.46	3.39			<0.01
	treatment (linear)	−0.36	−1.54			0.13
	treatment (quadratic)	−0.24	−1.00			0.32
	s(time point)			0.92	2.60	<0.001
	s(time point): low-dose polyI:C			0.00	0.00	0.35
	s(time point): high-dose polyI:C			0.14	0.04	0.19

Although our sample size precludes formal statistical investigation into pairwise hourly differences, body temperature was immediately elevated in the high-dose polyI:C group, while it initially decreased in the PBS and low-dose polyI:C groups (mean temperature change from pre-injection baseline at hour one = high-dose polyI:C: 0.23°C; low-dose polyI:C: −1.87°C; PBS: −1.93°C). The high- and low-dose polyI:C groups differed by an average of greater than 1°C only for the first 3 hours of the trial (3/24 hours); however, by hour 4 and throughout the remainder of the trial, the mean temperature difference between these groups was less than 1°C. By contrast, the high-dose polyI:C and PBS bats differed by an average of greater than 1°C for most of the trial (17/24 hours), including in hours 21–23.

Treatment did not affect body mass or total leucocyte counts (figure 1) independently or in interaction with time (table 1). These GAMMs explained 77% and 28% of the deviance and 72% and 23% of the variance, for mass and leucocyte counts, respectively. Repeatability was generally low, with among-individual variance only explaining 32% (intraclass correlation coefficient (ICC) = 0.32, 95% confidence interval (CI) = 0.15 to 0.65), 14% (ICC = 0.14, 95% CI = −0.02 to 0.51) and 4% (ICC = 0.04, 95% CI = −0.07 to 0.36) of total variation in temperature, mass and white blood cell counts, respectively.

Our post-hoc power analysis revealed that our study only had an 8% chance of correctly rejecting the null hypothesis. A sample size of 53 bats per treatment group would have been required to statistically detect moderate treatment effects.

## 4. Discussion

The acute phase response is associated with several sickness symptoms, including lethargy, fever and anorexia, that can help explain variation in infection outcomes, including morbidity and mortality, pathogen transmission and spillover. Because sickness symptoms are highly variable within and between species, characterizing the acute phase response across species and contexts can help to predict or mitigate these individual, population and community-level effects. These investigations are particularly interesting and epidemiologically relevant with bats, as many of these flying mammals often harbour pathogens—particularly viruses—that are dangerous to other animals, while seemingly remaining asymptomatic [8,9,57]. To improve understanding of how bats respond to viral challenges, we experimentally challenged wild phyllostomid bats with a viral mimic (i.e. polyI:C). Our findings demonstrate that polyI:C elicits an immune response *in vivo* in *C. perspicillata*. The primary indicator of this response was fever: immune-challenged bats had higher body temperatures compared to control bats over a 24-hour period. By contrast, immune-challenged bats did not differ from control bats in body mass or total leucocyte counts. These results remained the same regardless of the polyI:C dose administered (i.e. 2 versus 5 mg kg^−1^), emphasizing that either dose is appropriate for studying antiviral responses in this species. Given the small sample size (*n* = 9), our study is underpowered to statistically detect moderate effects, and our results therefore should be considered preliminary and interpreted with caution. Nevertheless, our findings improve understanding of how wild bats respond to viral infections and will help facilitate future work on studying the antiviral response in wild bats.

In our study, regardless of the polyI:C dose administered, challenged *C. perspicillata* exhibited increased body temperature. Between the high-dose and control bats, this temperature difference was greater than 1°C for 71% of the trial, revealing an energetic cost to *C. perspicillata*. Indeed, in *A. lituratus*, another bat species from the same family as *C. perspicillata,* a 1.2°C temperature increase following polyI:C challenge was associated with a 20% increase in resting metabolic rate [42]. However, whereas the closely related *A. lituratus* also develops fever [42], the much more distantly related *M. myotis* does not—even at much higher doses (i.e. 25 mg kg^−1^ compared to our highest dose of 5 mg kg^−1^) [41]. These results suggest that more closely related or more ecologically similar species could show similar febrile responses to viral challenges. However, methodological differences also point to the possibility that these discrepancies could be artefacts rather than shared ecology or evolutionary history. For instance, in addition to different doses, prior work on *A. lituratus* reported body temperature 6 hours after polyI:C treatment, whereas that on *M. myotis* reported body temperature 24 hours later [41,42]. As revealed in our study, febrile responses to polyI:C can vary in a time- and dose-dependent manner; as such, these sampling windows (6 versus 24 hours) might be comparatively incompatible. This last point emphasizes the value of fine-scale temporal sampling for characterizing febrile responses to polyI:C and stresses that future comparative work with wild bats necessitates standardizing protocols to the fullest extent possible, while acknowledging that different species might require different doses to elicit similar responses and that these responses might differ under varying conditions or timescales [58].

We did not observe any body mass or leucocyte count differences between polyI:C- and PBS-injected bats. It is possible that we simply did not have the statistical power to detect these effects. Yet these findings also could be biologically meaningful, as they are consistent with previous work on *M. myotis*, which also do not lose body mass or exhibit leucocytosis following polyI:C treatment [41]. Still, that study also found that the number of lymphocytes decreased in immune-challenged bats, highlighting that a lack of leucocytosis does not necessarily reflect the absence of a cellular response. Although we did not measure differential counts in the present study, this would be a relevant avenue for future work. In *A. lituratus,* although leucocytosis was not measured, bats injected with polyI:C lost approximately 1 g of body mass more than control bats after 6 hours, although this effect had seemingly dissipated by 24 hours post-treatment [42]. Variable body mass responses to viral challenges differ from many bacterial challenge studies, which are largely more consistent, at least for Neotropical bat species. Indeed, Neotropical bats, including but not limited to *C. perspicillata,* typically lose mass when challenged with LPS (e.g. [14–17,20–22,24,25]). Body mass loss following an immune challenge can be owing to decreased appetite or increased energy metabolism, both of which facilitate allocating resources towards fighting infection [4]. In our study, polyI:C challenged bats did not seem to experience appetite loss, given that—regardless of treatment group—all bats ate most, if not all, of the banana provided during the trial (we did not formally measure the amount of banana consumed). The mechanisms for why bats do not always lose mass following viral challenges remain unclear. Future comparative work could aim to determine the extent to which this effect is a feature of the bacterial versus viral response and by how much this variation can be explained by ecological (e.g. diet), evolutionary, or methodological (e.g. time in captivity, challenge dose) differences between individuals, species and studies.

Despite the lack of mass or leucocyte effects associated with polyI:C treatment, the febrile response in our study provides the practically useful information that as little as 2 mg kg^−1^ of polyI:C is sufficient to elicit an acute phase response *in vivo* in *C. perspicillata*. Similar results were observed with 5 mg kg^−1^ of polyI:C, with the timing of body temperature changes being the apparent difference between our two doses. Specifically, body temperature of the high-dose group (i.e. 5 mg kg^−1^) was seemingly elevated relative to the control group immediately, whereas temperature of the low-dose group (i.e. 2 mg kg^−1^) increased more gradually, converging with the high-dose group approximately 4 hours after treatment. Although these hourly pairwise comparisons were not validated statistically, and must therefore be interpreted cautiously, we highlight them to help inform the methodological decisions of researchers studying viral-associated responses in wild bats. In short, depending on the goals of a study and the timing of desired physiological effects, either dose could be suitable to administer to wild bats.

### 4.1. Future directions

This initial study was essential for determining that polyI:C elicits an immune response *in vivo* in *C. perspicillata*, an emerging model system for studies of bat–virus interactions (e.g. [44,45]). Although our analyses were underpowered to detect effects statistically, the consistent febrile response—regardless of dose—corroborates immune activation. With this response now validated, opportunities exist to expand on our work, continuing to improve understanding of wild bat antiviral responses and variation in the acute phase response more generally across taxa. An obvious goal for future work should be validating the trends we observed here, and this objective could be achieved in parallel to testing hypotheses about other drivers of sickness variation.

Variation in sickness responses could emerge at several levels of biological organization, and *in vivo* polyI:C challenges with wild *C. perspicillata* could test these hypotheses at varying scales. Within individuals, for instance, sickness could change throughout ontogeny or in response to shifting environmental conditions (e.g. across seasons). Between individuals, responses could vary with factors such as infection status, age or sex. In our study, because most female bats were pregnant at the time of sampling, we only investigated males. Given that bat antiviral responses have received attention for possible human medical implications, characterizing sex effects should be prioritized. Among populations, polyI:C challenges with *C. perspicillata* captured in diverse environments (e.g. along naturally occurring gradients, from range edges versus cores or from habitats experiencing differing levels of human disturbance) could provide insight into how ecological or evolutionary factors contribute to this variation [59]. Findings from this future work on *C. perspicillata* specifically could then be extended to make among-species comparisons, testing how phylogenetic relationships or life-history variation shape antiviral sickness responses, improving understanding of how this variation affects individual fitness, evolutionary trajectories and ecological processes including pathogen transmission and spillover [26,60,61].

In testing these ideas, researchers could expand our methods by investigating other facets of physiology and immunology. For instance, LPS research has considered specific acute phase proteins (e.g. haptoglobin), bacterial killing ability, and indicators of oxidative stress (e.g. [39,41,62]). These measures could also be hypothesized to shift following polyI:C treatment. More mechanistic insight into antiviral responses could also be gained by using transcriptomics or proteomics to characterize broader immune gene expression or protein abundance, respectively [63]. To develop a more holistic view of antiviral responses, researchers could also investigate interactions between immunity and other biological systems. For instance, the microbiome interacts with the immune system to impact host health, and the acute phase response initiated by LPS shifts bat gut microbiome communities [62]. Although these examples are non-exhaustive, the scarcity of *in vivo* challenge research with viral mimics in wildlife presents substantial opportunities for future work.

### 4.2. Conclusions

Our work therefore reveals that while *C. perspicillata* does not show all the classically expected components of the acute phase response to viral challenge, a febrile response does develop following subcutaneous injection with polyI:C. Discussing our findings in the context of other bat immunology research emphasizes that study-level and species-level variation in the acute phase response is the norm. More widespread challenge trials with viral mimics are needed in wild bats and other animals to untangle the sources and extent of this variation and its ecological and evolutionary implications. Our study provides practically useful information on the dosage and timing of the acute phase response initiated by polyI:C injection in one of the most studied Neotropical bat species. Our findings are therefore useful to guide this future work for researchers interested in making comparisons within or across species and for designing studies investigating how the acute phase response affects, or is affected by, other behavioural or physiological processes. More broadly, some bats harbour viruses that cause disease in other animals while presenting as asymptomatic, yet most past work with these flying mammals has focused on their responses to bacterial antigens. In monitoring fine-scale physiological changes following challenge with a viral mimic, our study begins to fill an important knowledge gap in how wild bats respond to viral infections and contributes to a growing body of work aiming to understand the causes and consequences of sickness across wildlife.

## Supplementary Material



## Data Availability

The data and code necessary to replicate these findings are available as supplementary material [64].
