# Supplementary material for: A viral mimic increases body temperature but does not affect mass or inflammation in a wild frugivorous bat
Source: R Soc Open Sci. 2026 Nov 11;13(11):262283. doi: 10.1098/rsos.262283 (PMC13750927; doi:10.1098/rsos.262283)
Supplement: Supplementary file 1 [file rsos.262283.zip › rsos.262283/rsos262283_si_004.docx]

**Supplementary materials for: A viral mimic increases body temperature but does not affect mass or inflammation in a wild frugivorous bat**

Table S1. Basis dimension diagnostics for the smooth terms (excluding random effects terms) in each model. For each model, all smooth terms were fit using an initial k = 7.

| Model | Term | k' | EDF | k-index | p |
| --- | --- | --- | --- | --- | --- |
| Body temperature | s(timePoint) | 6 | 1.391 | 0.655 | <0.001 |
|  | s(time point) : low-dose polyI:C | 6 | 2.497 | 0.655 | <0.001 |
|  | s(time point) : high-dose polyI:C | 6 | 0.395 | 0.655 | <0.001 |
| Body mass | s(timePoint) | 6 | 4.028 | 0.949 | 0.318 |
|  | s(time point) : low-dose polyI:C | 6 | 0.000 | 0.949 | 0.308 |
|  | s(time point) : high-dose polyI:C | 6 | 0.000 | 0.949 | 0.280 |
| Leukocyte count | s(timePoint) | 6 | 0.919 | 0.961 | 0.368 |
|  | s(time point) : low-dose polyI:C | 6 | 0.000 | 0.961 | 0.373 |
|  | s(time point) : high-dose polyI:C | 6 | 0.143 | 0.961 | 0.295 |

Figure S1. Effects of treatment on raw body temperature (°C) over 24 hours. ‘Low’ and ‘high’ treatment refers to the polyI:C dosage.

Figure S2. Effects of treatment on raw body mass (g) over 24 hours. ‘Low’ and ‘high’ treatment refers to the polyI:C dosage.

Figure S3. Effects of treatment on raw leukocyte counts over 24 hours. ‘Low’ and ‘high’ treatment refers to the polyI:C dosage.

Figure S4. Diagnostic plots for the temperature GAMM.

Figure S5. Diagnostic plots for the mass GAMM.

Figure S6. Diagnostic plots for the leukocyte count GAMM.
